# A comparison of three accelerometry-based devices for estimating energy expenditure in adults and children with cerebral palsy

**DOI:** 10.1186/1743-0003-11-116

**Published:** 2014-08-05

**Authors:** Jennifer M Ryan, Michael Walsh, John Gormley

**Affiliations:** 1 School of Medicine, Trinity College Dublin, Dublin, Ireland; 2 Gait Laboratory, Central Remedial Clinic, Clontarf, Dublin, Ireland; 3 Centre for Research in Rehabilitation, Brunel University, London, UK

**Keywords:** Assessment, Physiotherapy, Rehabilitation, Sedentary living, Exercise

## Abstract

**Background:**

Advanced accelerometry-based devices have the potential to improve the measurement of everyday energy expenditure (EE) in people with cerebral palsy (CP). The aim of this study was to investigate the ability of two such devices (the Sensewear ProArmband and the Intelligent Device for Energy Expenditure and Activity) and the ability of a traditional accelerometer (the RT3) to estimate EE in adults and children with CP.

**Methods:**

Adults (n = 18; age 31.9 ± 9.5 yr) and children (n = 18; age 11.4 ± 3.2 yr) with CP (GMFCS levels I-III) participated in this study. Oxygen uptake, measured by the Oxycon Mobile portable indirect calorimeter, was converted into EE using Weir’s equation and used as the criterion measure. Participants’ EE was measured simultaneously with the indirect calorimeter and three accelerometers while they rested for 10 minutes in a supine position, walked overground at a maximal effort for 6 minutes, and completed four treadmill activities for 5 minutes each at speeds of 1.0 km.h^−1^, 1.0 km.h^−1^ at 5% incline, 2.0 km.h^−1^, and 4.0 km.h^−1^.

**Results:**

In adults the mean absolute percentage error was smallest for the IDEEA, ranging from 8.4% to 24.5% for individual activities (mean 16.3%). In children the mean absolute percentage error was smallest for the SWA, ranging from 0.9% to 23.0% for individual activities (mean 12.4%). Limits of agreement revealed that the RT3 provided the best agreement with the indirect calorimeter for adults and children. The upper and lower limits of agreement for adults were 3.18 kcal.min^−1^ (95% CI = 2.66 to 3.70 kcal.min^−1^) and -2.47 kcal.min^−1^ (95% CI = -1.95 to -3.00 kcal.min^−1^), respectively. For children, the upper and lower limits of agreement were 1.91 kcal.min^−1^ (1.64 to 2.19 kcal.min^−1^) and -0.92 kcal.min^−1^ (95% CI = -1.20 to -0.64 kcal.min^−1^) respectively. These limits of agreement represent -67.2% to 86.3% of mean EE for adults and -36.5% to 76.3% of mean EE for children, respectively.

**Conclusions:**

Although the RT3 provided the best agreement with the indirect calorimeter the RT3 could significantly overestimate or underestimate individual estimates of EE. The development of CP-specific algorithms may improve the ability of these devices to estimate EE in this population.

## Background

Cerebral palsy (CP) is defined as ‘a group of permanent disorders causing activity limitation that are attributed to non-progressive disturbances that occurred in the developing foetal or infant brain’ [1]. CP is the most common form of childhood disability, occurring in approximately 2 to 3 per 1000 live births [2]. Although considered a paediatric condition, the majority of children with CP will live well into adulthood [3]. The motor impairments associated with CP result in an increased energy cost of locomotion compared to able-bodied people [4,5]. This increased energy requirement is associated with difficulties in performing everyday tasks [6] and low levels of habitual physical activity [7]. Physical inactivity feeds into a negative cycle of reduced levels of cardiorespiratory fitness [8], increased physical strain associated with walking [5,9] and functional deterioration [10,11], leading to further inactivity. Interventions such as surgery and exercise programmes can improve cardiorespiratory fitness, gait efficiency and gross motor function among people with CP [12,13]. Further research is required however, to investigate if these improvements carry over to increased levels of habitual physical activity. To accurately assess habitual physical activity in people with CP validated and feasible measurement tools are required. Few validated measures currently exist [14].

Accelerometry-based devices have been used to measure habitual physical activity in large population-based studies [15,16]. Although they have been validated for use in different segments of the population little has been done to test their use in adults and children with CP. Traditionally, accelerometers are worn on the hip and measure the magnitude of the body’s acceleration, as indicated by the ‘count’ output. Physical activity intensity can be categorised as sedentary, light, moderate or vigorous according to the value of counts per minute. More recently some manufacturers have included inbuilt proprietary algorithms to convert counts into energy expenditure (EE) [typically in kilocalories (kcal)]. Although by providing a direct output of EE accelerometers may be more accessible and easy to use, particularly if marketed towards the general population, the ability of an accelerometer to provide a point estimate of EE from limited information is questionable [17].

Recent advances in technology and modelling techniques have led to the development of new pattern recognition devices that provide alternative ways of measuring and evaluating physical activity. These advanced accelerometry-based devices combine inputs from multiple sources, which may improve their ability to accurately estimate EE, compared to traditional accelerometers. In particular, by combining data from multiple sources, they have the potential to improve the estimation of EE in people with CP, whose biomechanical efficiency is different to that of able-bodied people.

The Sensewear Pro Armband (SWA) (Bodymedia, Inc.) and the Intelligent Device for Energy Expenditure and Activity (IDEEA) (Minisun, LLC) are two such devices. The SWA is a multisensor device, worn on the upper arm that combines accelerometry data with information from several heat-related channels to estimate EE using proprietary algorithms. The SWA provides more accurate estimates of EE than traditional accelerometers in able-bodied adults and children [18,19]. The IDEEA collects raw data from five accelerometer sensors, which are attached to the chest, anterior aspects of both thighs and soles of the feet, and converts it to EE using proprietary algorithms. The ability of the IDEEA to detect 35 postures and define temporal-spatial gait parameters sets it apart from other accelerometers.

To date, the ability of the IDEEA, and a traditional accelerometer, the Actigraph 7164, to estimate EE in children with CP has been investigated [20-22]. No study, however, has investigated the validity of accelerometry-based devices in adults with CP. Furthermore, the ability of advanced multisensor devices to estimate EE in people with CP has not been directly compared to that of traditional accelerometers. Therefore, it is unknown if newly developed technology represents an improvement upon existing devices and technology. The present study aimed to evaluate the validity of advanced accelerometry-based devices (the SWA and the IDEEA) at estimating EE in adults and children with CP, compared to a traditional accelerometer (the RT3). It was hypothesised that the advanced, multisensor devices would more accurately estimate EE compared to the traditional accelerometer.

## Method

### Participants

Ambulant children (≥6 years) and adults (≥18 years) with a medically confirmed diagnosis of CP were recruited for this study through a national centre that provides services for adults and children with disabilities. Individuals with a severe cognitive impairment, uncontrolled epilepsy or seizure activity, or an acute lower limb injury were excluded from participating. Physiotherapists provided seventy-nine eligible participants with information about the study over a period of nine months. Thirty-six people agreed to participate in the study.

Participants were predominantly male (10 men and 10 boys). Participants were classified as level I, II or III on the Gross Motor Function Classification System (GMFCS) by their physiotherapist [23]. The GMFCS is a classification system that allows people with cerebral palsy to be classified according to their level of functional mobility and use of mobility aids. People in level I can walk and run independently but may have difficulty with coordination or speed. People in level II can walk independently but may have difficulty running. People in level III require a mobility aid to walk independently and may use wheeled mobility to travel long distances. Participants’ characteristics across GMFCS level are presented in Table 1. Fifteen adults (83%) and 13 children (72%) used no ambulatory aid. One adult walked with a 3-wheeled rollator, one adult walked with a stick, one adult and two children walked with 2 elbow crutches and three children walked with the aid of a K-walker. Seven children (39%) and ten adults (56%) had bilateral spastic CP; the remaining participants had unilateral spastic CP.

**Table 1:** Characteristics of adults and children across levels of Gross Motor Function Classification System (GMFCS)

	**Adults**				**Children**			
	All	GMFCS	GMFCS II	GMFCS	All	GMFCS I	GMFCS II	GMFCS
	(n = 18)	I (n = 9)	(n = 7)	III (n = 2)	(n = 18)	(n = 10)	(n = 4)	III (n = 4)
Age (yr)	31.9 ± 9.5	28.1 ± 7.8	34.9 ± 10.7	39.0 ± 8.5	11.4 ± 3.2	11.5 ± 3.8	10.0 ± 2.2	12.5 ± 1.9
Weight (kg)	68.2 ± 13.5	69.0 ± 13.3	67.8 ± 16.2	65.8 ± 10.2	44.6 ± 16.9	46.5 ± 20.9	37.0 ± 12.0	47.3 ± 8.2
Height (cm)	163.9 ± 10.3	166.6 ± 9.3	162.0 ± 12.2	158.5 ± 9.2	147.0 ± 18.5	149.5 ± 21.1	140.0 ± 20.1	147.6 ± 10.3
BMI (kg.m^−2^)	25.3 ± 4.8	24.6 ± 3.8	25.9 ± 5.9	26.6 ± 7.1	20.0 ± 4.5	20.0 ± 5.2	18.5 ± 1.8	21.9 ± 4.7

All participants completed the Physical Activity Readiness Questionnaire to screen for conditions contraindicating participation in exercise. The procedures and risks involved in the study were fully explained to participants and their guardians (in the case of participants less than 18 years of age or with a mild-to-moderate intellectual disability). Written informed consent was provided before testing proceeded. Ethical approval for this study was granted by the Faculty of Health Sciences and the Central Remedial Clinic's ethics committee.

### Instrumentation

#### RT3 accelerometer

The RT3 (Stayhealthy Inc.) is a small (7.1 × 5.6 × 2.8 cm), lightweight, unobtrusive device that is worn on the right hip in the midaxillary line. The device consists of a piezoelectric element and a seismic mass which generate a variable output voltage signal when the participant moves. The size of the voltage is proportional to the applied acceleration. The voltage is filtered, amplified and sampled at a rate of 1 Hz to convert the voltage signal to a series of numbers called counts. The piezoelectric element is sensitive to accelerations in three dimensions i.e. the vertical plane (x), the antero-posterior plane (y) and the medio-lateral plane (z). A resulting vector magnitude (VM) is calculated as the square root of the sum of squared activity counts for each dimension. Inbuilt proprietary algorithms convert count data into total EE based on age, sex, height and weight.

### Sensewear Pro Armband

The SWA (Bodymedia, Inc.) is a lightweight (83 g) monitor that is worn on the right arm over the triceps muscle at the midpoint between the acromion and the olecranon. It combines accelerometry data, heat loss data, skin temperature and galvanic skin response data with information about participants’ sex, age, height and weight to predict EE with the use of inbuilt algorithms. Data were processed using Sensewear Software version 6.1.

### Intelligent Device for Energy Expenditure and Activity

The IDEEA (Minisun, LLC) consists of five sensors, which are attached to the chest, thighs, and soles of the feet, that collect data and transmit it through thin, flexible wires to the recorder. Before each test the device is calibrated to ensure correct placement of the sensors. The IDEEA provides information regarding the type, duration, and estimated EE of each activity carried out by the user while wearing the device. EE is estimated using inbuilt algorithms, which incorporate information about age, sex, weight, height and a subjective estimation of fitness level (on a scale of 1 - 10) with accelerometry data.

### Oxycon Mobile indirect calorimeter

Oxygen uptake, measured by the Oxycon Mobile portable indirect calorimeter (IC) (Carefusion Germany 234 GmBh, Hoechberg, Germany), was converted into EE using Weir’s equation [24]. The Oxycon has been shown to be an accurate measure of oxygen uptake [25] and has been used previously as a criterion measure of EE in children and adults [26,27]. It consists of a soft, flexible facemask and an analyser unit (950 g) that is attached to a chest harness worn by the participant. Expired air is channeled through a bidirectional digital volume sensor. Gas concentrations are collected with a Nafion sampling tube. Participants also wore a Polar heart-rate monitor throughout the test. Gas, flow and heart-rate data were sent telemetrically to the calibration and receiver unit, which is connected to a personal computer before being processed in the PC-software (JLAB, Carefusion Germany 234 GmbH, Hoechberg, Germany). Volume calibration, ambient gas calibration and reference gas calibration (reference gas tank: 16% O_2_, 5% CO_2_) were performed immediately prior to each test using the built-in automated procedures.

### Protocol

Participants attended a physiotherapy gym on one occasion where their height to the nearest 0.5 cm, and weight to the nearest 0.1 kg, (SECA, digital scales) were measured. The three accelerometers and the IC were configured for each participant using their anthropometric and demographic details, and attached to each participant. In the case of significant asymmetry the RT3 and SWA were attached to the least affected side. Energy expenditure data were collected using the IC and each monitor during rest and a number of walking activities. Walking activities were selected following pilot testing to represent locomotor activity that covered a variety of intensities while still being safe for participants to complete. Participants’ initially lay in supine position for 10 min while resting energy expenditure data were collected. They then completed a 6 minute walking trial on a 70 m corridor at maximal effort. Following this, participants were given a 5 min familiarisation period with the treadmill. They then walked on a calibrated treadmill at speeds of 1.0 km.h^-1^ at 0% incline, 1.0 km.h^−1^ at 5% incline, 2.0 km.h^−1^ at 0% incline, and 4.0 km.h^−1^ at 0% incline. Children did not complete treadmill walking at 4.0 km.h^−1^. Participants walked for 5 minutes at each speed in the order presented. Participants rested in a seated position between each activity until their heart-rate and oxygen consumption returned to baseline values. Due to the variation in gross motor function between participants not all participants completed all treadmill activities: 14 adults completed treadmill walking at 1.0 km.h^−1^, 1.0 km.h^−1^ at 5% incline, and 2.0 km.h^−1^, respectively; 9 adults completed treadmill walking at 4.0 km.h^−1^; 15, 13 and 11 children, respectively, completed treadmill walking at 1.0 km.h^−1^, 1.0 km.h^−1^ at a 5% incline, and 2.0 km.h^−1^.

### Data processing

EE from the IC was observed in 30-s epochs. Data from the SWA and RT3 were recorded in 1-min epochs. Data from the IDEEA were observed in 1-s epochs. The IC and each monitor was synchronised with a single laptop clock. Exact start and stop times of each activity were recorded from this clock. Following completion of the protocol data were downloaded from the three monitors. Data were examined visually to check for malfunctioning units, time synchronisation and abnormal outputs.

One child’s overground walking data and one adult’s treadmill walking data at 1.0 km.h^−1^ at 5% incline were removed because of a problem with the IC during these activities. RT3 data from one adult and IDEEA data from two adults were missing because of equipment malfunction. One child’s SWA data for treadmill walking at 2.0 km.h^−1^ and one adult’s SWA data for treadmill walking at 1.0 km.h^−1^ and 1.0 km.h^−1^ at 5% incline were removed because of abnormal data obtained during these activities. Final sample sizes ranged from n = 8 to n = 18 for adults and n = 10 to n = 18 for children. The final 2 min of EE data (kcal.min^−1^) from each activity (supine lying and walking activities) were extracted and averaged over the 2 min period. Mean EE, in kcal.min^−1^, from each activity was used in statistical analysis.

### Statistical Analysis

Statistical analysis was performed using Analyse-It for Microsoft Excel, version 2.26 and SPSS, version 20. Statistical significance was set at p < 0.05. Descriptive variables are presented as means and standard deviation. A one-way repeated measures ANOVA was used to detect differences in EE between methods. Post-hoc analyses using paired t-tests with the Bonferroni correction were conducted to examine specific differences in EE between each monitor and the IC. The mean absolute percentage error was calculated for individual activities based on the absolute value of the individual errors. This method reflects the true error in estimation and provides the most appropriate indicator of overall error.

Further analyses were conducted to examine the level of agreement between measures. Bland-Altman plots [28] were calculated to examine the level of agreement between each monitor and the IC across the range of activities. Limits of agreement were calculated as ±2 SD from the overall mean bias between the IC and each monitor. The limits of agreement are presented as kcal.min^−1^ and in a percentage of the mean EE between the IC and each respective monitor.

## Results

### Differences in energy expenditure between the indirect calorimeter and each monitor

Repeated measures ANOVA revealed a significant monitor effect on EE for all activities except for overground walking in children (Table 2). Post-hoc analyses for adults revealed that the SWA significantly underestimated resting EE and overestimated EE during treadmill walking at 1.0 km.h^−1^ at 0% and 5% incline; the RT3 underestimated EE during treadmill walking at 1.0 km.h^−1^ at 0% and at 5% incline; the IDEEA underestimated EE during overground walking (p < 0.01 for all). In children, the SWA underestimated resting EE and the RT3 underestimated EE for all treadmill activities (p < 0.01 for all).

**Table 2:** **Mean energy expenditure for each activity and mean difference (kcal.min**^**−1**^**) between methods (kcal.min**^**−1**^**)**

	**Speed**	**IC**	**SWA**	**IC-SWA**	**RT3**	**IC-RT3**	**IDEEA**	**IC-IDEEA**
Adults								
Rest	NA	1.27 ± 0.28	1.09 ± 0.15	0.19 ± 0.27*	1.21 ± 0.16	0.04 ± 0.24	1.07 ± 0.45	0.22 ± 0.42
Walk (overground)	4.2 ± 1.2 km.h^−1^	6.69 ± 2.04	6.88 ± 1.60	−0.19 ± 2.24	7.52 ± 2.25	−0.85 ± 2.32	5.04 ± 1.14	1.78 ± 2.22*
Treadmill walking	1.0 km.h^−1^	3.14 ± 0.84	5.32 ± 2.48	−2.13 ± 1.97*	2.14 ± 0.54	0.99 ± 0.48*	2.72 ± 1.17	0.40 ± 1.05
Treadmill walking	1.0 km.h^−1^ at 5% incline	3.45 ± 0.85	6.09 ± 2.72	−2.64 ± 2.19*	2.08 ± 0.43	1.38 ± 0.67*	2.59 ± 1.09	0.91 ± 1.06
Treadmill walking	2.0 km.h^−1^	3.90 ± 0.93	5.28 ± 2.89	−1.38 ± 2.64	3.13 ± 0.96	0.77 ± 1.18	3.22 ± 0.80	0.78 ± 1.07
Treadmill walking	4.0 km.h^−1^	5.10 ± 1.21	6.07 ± 1.97	−0.89 ± 2.09	4.98 ± 0.93	0.12 ± 0.83	4.48 ± 1.48	0.62 ± 1.90
Children								
Rest	NA	1.06 ± 0.33	0.79 ± 0.28	0.28 ± 0.30*	1.03 ± 0.19	0.04 ± 0.25	1.49 ± 0.81	−0.43 ± 0.76
Walk (overground)	3.6 ± 1.3 km.h^−1^	4.56 ± 1.47	4.39 ± 1.89	0.17 ± 1.54	4.33 ± 1.64	0.23 ± 0.92	3.92 ± 1.46	0.64 ± 1.74
Treadmill walking	1.0 km.h^−1^	2.52 ± 0.82	2.67 ± 1.00	−0.03 ± 0.71	1.79 ± 0.46	0.73 ± 0.47*	2.50 ± 0.58	0.02 ± 0.78
Treadmill walking	1.0 km.h^−1^ at 5% incline	2.73 ± 1.08	3.50 ± 2.04	−0.63 ± 1.43	1.86 ± 0.52	0.87 ± 0.61*	2.32 ± 0.91	0.41 ± 1.25
Treadmill walking	2.0 km.h^−1^	3.11 ± 1.29	3.57 ± 1.56	−0.28 ± 1.31	2.20 ± 0.71	0.91 ± 0.70*	2.83 ± 0.69	0.28 ± 1.10

*p<0.01.

IC, indirect calorimeter.

**Figure 1:**
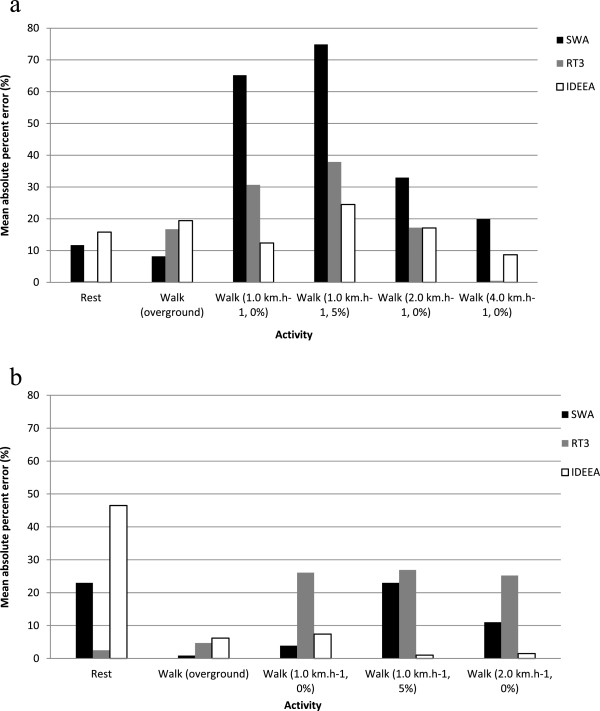
Mean absolute percentage error of the SWA, RT3 and IDEEA for (a) adults and (b) children.

In adults, the mean absolute percentage error for individual activities ranged from 8.2% to 74.9% for the SWA (mean 35.5%), from 0.4% to 37.9% for the RT3 (mean 17.2%), and from 8.4% to 24.5% for the IDEEA (mean 16.3%). The errors in EE for the SWA and the RT3 were largest for treadmill walking at 1.0 km.h^−1^ at 0% incline and at 5% incline. Errors in EE estimates from the IDEEA did not vary considerably across activities (Figure 1a).

In children, the mean absolute percentage error for individual activities ranged from 0.9% to 23.0% for the SWA (mean 12.4%), from 2.5% to 26.9% for the RT3 (mean 17.0%), and from 1.0% to 46.5% for the IDEEA (mean 12.5%) (Figure 1b). The error in EE estimation from the IDEEA was much larger for rest compared to locomotor activities. This may be attributed to an extreme value of 4.41 kcal.min^−1^ recorded by the IDEEA for one child. When this was removed the mean absolute percentage error reduced from 46.5% to 36.2% for rest, and from 12.5% to 10.5% for all activities combined.

### Agreement in energy expenditure between the indirect calorimeter and each monitor

**Figure 2:**
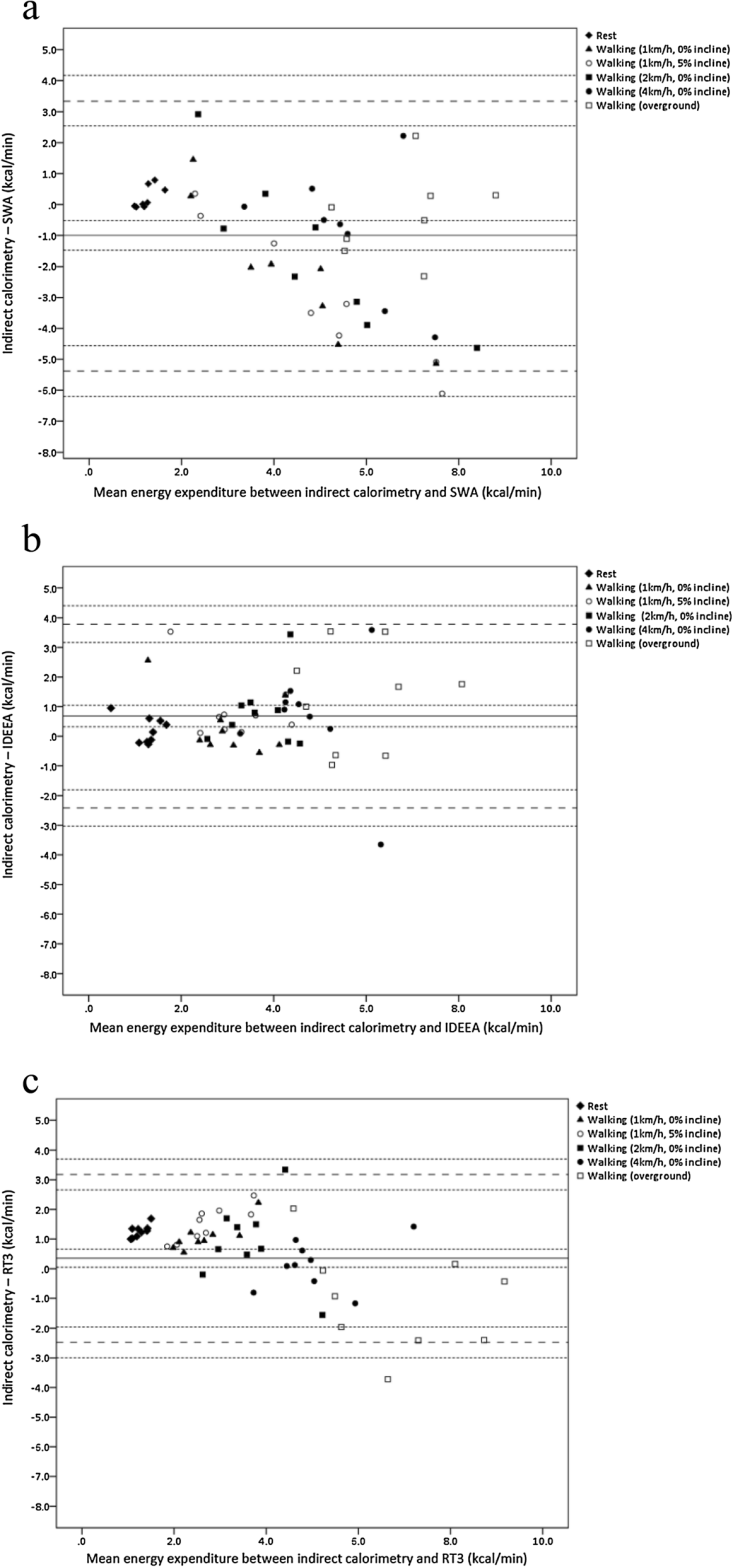
**Bland-Altman plots between energy expenditure from the indirect calorimeter (IC) and accelerometry-based devices for adults.** The middle solid lines represent the mean difference between the methods for parts **a)** IC vs. SWA, **b)** IC vs. IDEEA, and **c)** IC vs. RT3. The wide dashed lines represent the upper and lower limits of agreement. The narrow dashed lines represent the 95% confidence intervals.

**Figure 3:**
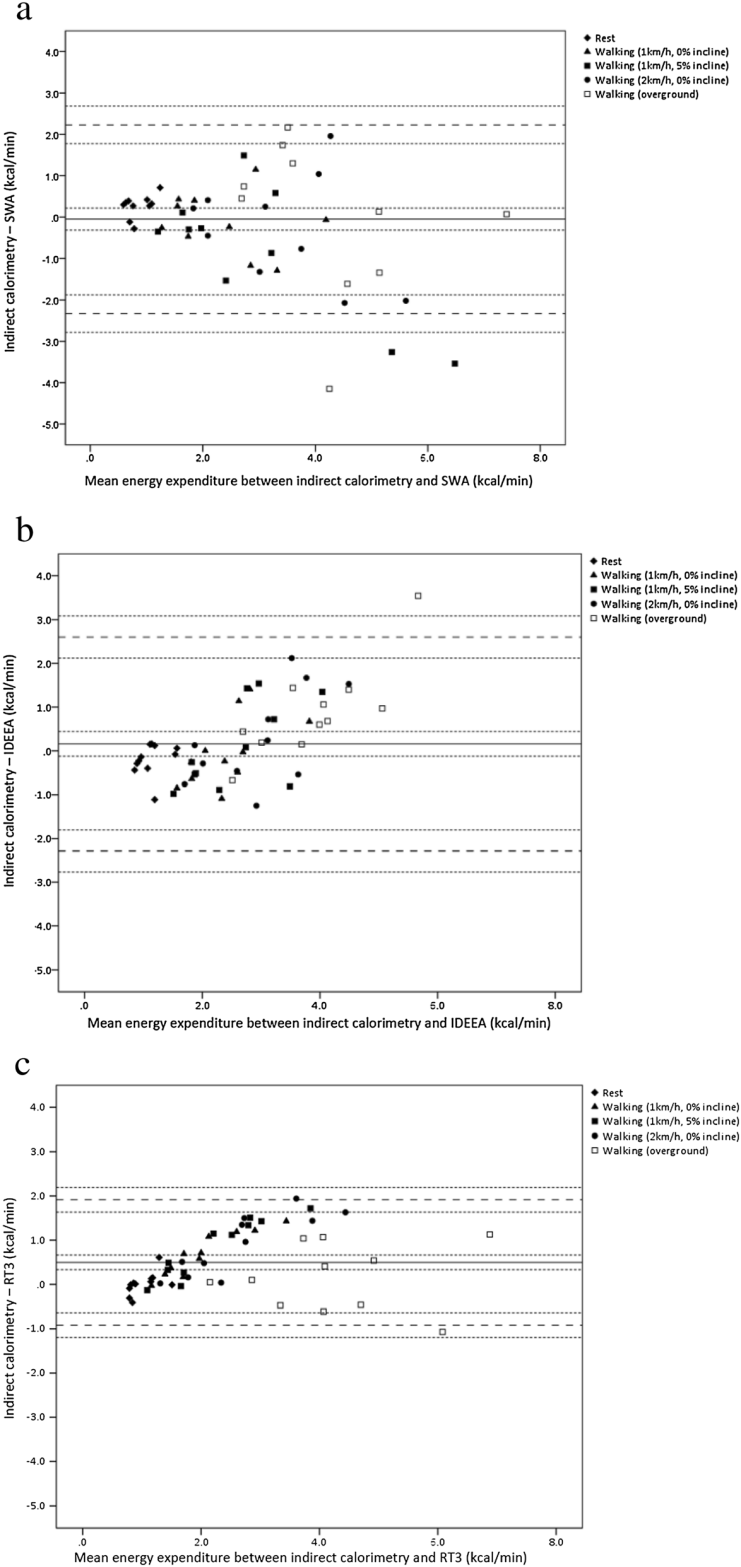
**Bland-Altman plots between energy expenditure from the indirect calorimeter (IC) and accelerometry-based devices for children.** The middle solid lines represent the mean difference between the methods for parts **a)** IC vs. SWA, **b)** IC vs. IDEEA, and **c)** IC vs. RT3. The wide dashed lines represent the upper and lower limits of agreement. The narrow dashed lines represent the 95% confidence intervals.

In adults the mean bias and limits of agreement were smallest for the RT3 (Figure 2c). The mean bias was 0.35 kcal.min^−1^(95% CI = 0.05 to 0.66 kcal.min^−1^) and the lower and upper limits of agreement were -2.47 kcal.min^−1^ (95% CI = -3.00 to -1.95 kcal.min^−1^) and 3.18 kcal.min^−1^ (95% CI = 2.66 to 3.70 kcal.min^−1^), respectively. The limits of agreement represented -67.2% (95% CI = -81.3% to -53.0%) to 86.3% (95% CI = 72.2% to 100.4%) of mean EE. The limits of agreement for the SWA and IDEEA were -5.38 to 3.35 kcal.min^−1^ (-123.0% to 76.7% of mean EE) and -2.41 to 3.78 kcal.min^−1^ (-65.1% to 101.9% of mean EE), respectively (Figure 2a and 2b, respectively). In children the SWA demonstrated the smallest mean bias (-0.05 kcal.min^−1^, 95% CI = -0.32 to 0.22 kcal.min^−1^) (Figure 3a). The limits of agreement, however, were narrowest for the RT3 [lower limit of agreement = -0.92 kcal.min^−1^ (95% CI = -1.12 to -0.64 kcal.min^−1^); upper limit of agreement = 1.91 kcal.min^−1^ (95% CI = 1.64 to 2.19 kcal.min^−1^)] (Figure 3c). These limits represented -36.5% (95% CI = -47.7% to -25.4%) to 76.3% (95% CI = 65.1% to 87.4%) of mean EE. The limits of agreement for the SWA and IDEEA were -2.33 to 2.23 kcal.min^−1^ (-83.1% to 79.6% of mean EE) and -2.28 to 2.60 kcal.min^−1^ (-85.3% to 97.1% of mean EE), respectively (Figure 3a and 3b, respectively).

## Discussion

The purpose of this study was to evaluate the validity of accelerometry-based devices at estimating EE in adults and children with CP. Although advanced accelerometry-based devices were hypothesised to be more accurate at estimating EE than a traditional accelerometer, the RT3 accelerometer demonstrated better agreement with the criterion measure. Despite this there was large inter-individual variation in estimates of EE, with limits of agreement ranging from -67.2% to 86.3% of mean EE for adults, and from -36.5% to 76.3% of mean EE for children.

The RT3 underestimated EE for two out of six activities for adults and three out of five activities for children. As expected, and in agreement with research in able-bodied adults and children [18,29-31], the RT3 was unable to detect the increased energy requirement of walking on a slope. The error in EE estimation from the RT3 for individual activities ranged from -37.9% to +16.7% for adults, and from -26.9% to -2.5% for children, implying that caution must be taken when using the RT3 to estimate EE. Validation studies of the RT3 in able-bodied adults have reported that the RT3 overestimated EE by 21% to 25% during ambulatory activities on level ground [32]. Overestimations of up to 108% have been observed for typically developing children walking on level ground [18,30]; no study however, reported the mean absolute percentage error. It is possible that the increased energy cost of locomotion associated with CP [4,5] counteracted the tendency for the RT3 to overestimate the EE of locomotion in able-bodied people, reducing the magnitude of the error in overestimation or resulting in an underestimation for some activities.

In contrast to the results of the current study, the SWA provided the best estimation of EE in adults and children without CP [18]. The SWA, however, overestimated EE for three out of five activities for children with CP and five out of six activities for adults with CP. Although an overestimation of EE may be counterintuitive, considering the higher metabolic cost of walking that’s associated with CP, it is possibly due to adaptations made to arm-swing in order to compensate for paresis. In children with unilateral CP, arm-swing on the least affected side is over 50% larger than the arm-swing of typically developing children [33]. Children with bilateral CP also increase their arm-swing length when attempting to increase walking speed, to compensate for the inability to increase leg-swing length [33]. Validation studies of the SWA in adults with multiple sclerosis and in adults with chronic stroke reported similar overestimations of EE during ambulation [34,35].

Of note, errors were smaller for the SWA and RT3 for overground walking, compared to treadmill walking, for both adults and children. The metabolic cost of walking is higher during treadmill walking than overground walking, for matched speeds, in adults with hemiparesis following stroke [36]. It is possible that participants’ metabolic cost and biomechanics altered during treadmill walking resulting in larger errors between the monitors and the IC. As treadmill walking speed increased the error reduced for both the SWA and RT3 for adults. This may be because only adults with minimal impairments were able to complete the higher treadmill speeds. Eight out of nine of the adults who completed treadmill walking at 4.0 km.h^−1^ were in GMFCS level I. It is likely that the monitors are more accurate among people in GMFCS level I as they have more efficient gait than people in GMFCS level II and III [6]. Errors did not similarly reduce as the treadmill speed increased in children, possibly because the sample did not become heterogeneous as the speed increased; children in GMFCS levels I, II and III completed treadmill walking at 2.0 km.h^−1^. The small sample size did not allow for subgroup analysis across levels of gross motor function. Future studies however should evaluate these monitors in each GMFCS level independently.

An essential problem with accelerometers is that there is a large variation in oxygen uptake among individuals for a given activity. The in-built equations used by these devices to estimate EE do not account for this variation and therefore are unable to provide accurate individual estimates of energy expenditure [18]. Additionally, these equations which are developed in the general population are unlikely to account for the increased energy cost of locomotion that is evident among people with CP and the significant variation in metabolic cost among people with CP [6]. This variation may contribute to the inaccuracy of individual estimates of EE observed in the current study. Calibration of CP-specific algorithms may improve the ability of these devices to estimate EE in this population. However, unless algorithms are developed for each GMFCS level a single CP-specific algorithm is unlikely to account for the variation in gait efficiency within the population.

To date, no study has validated accelerometers in adults with CP and only three have evaluated their validity in children. Two studies found that a hip-worn, traditional accelerometer (the Actigraph 7164) was an acceptable method of measuring moderate-to-vigorous physical activity in children with CP [21,22]. As this device was not simultaneously compared with other accelerometers its performance in comparison to other devices in children with CP is unknown. Future studies should compare the count output of accelerometers to provide an insight into which monitor might be most appropriate to use in this population. A previous study reported correlation coefficients of 0.70-0.88 between EE from the IDEEA and EE from an IC [20]. Although this suggests that the EE output from the two methods is related it does not suggest that they can be used interchangeably [37].

The inaccuracy of the IDEEA in the current study may be due to abnormal values been recorded for one child at rest (4.41 kcal.min^−1^) and two adults at rest (both 0 kcal.min^−1^). Values of 0 kcal.min^−1^ were also recorded for one adult walking at 1.0 km.h^−1^ at 0% and at 5% incline. No explanation could be provided for these abnormal values, and normal values were recorded for these participants during the remaining activities. These values were therefore included in the analysis. Previous studies have reported obtaining extreme values from the IDEEA, with no explanation for them [18,27]. Although removing these values may improve the accuracy of the IDEEA in the current study, validity is inextricably linked to reliability. The IDEEA needs to be consistently accurate if it is to be considered an acceptable measure of EE.

A strength of this study was the use of a reference method of oxygen uptake, which allowed the criterion validity as well as the concurrent validity of the monitors to be evaluated. Another strength was the use of a standardised protocol of locomotor activities, which facilitated comparison with a number of validation studies of the RT3, SWA and IDEEA in able-bodied adults and children. Although this study provides a comprehensive evaluation of the validity of these monitors during locomotor activity, caution should be used if drawing conclusions about the performance of these monitors during free-living activity. There are a number of limitations to this study such as the small sample size which may not have been sufficient to detect significant differences between monitors and the IC. The small sample size also resulted in poor precision of the estimated limits of agreement. However the 95% confidence intervals suggest that even on the most optimistic interpretation, in adults and children respectively, the RT3 can underestimate EE by 53.0% and 25.4% or overestimate EE by 65.1% and 72.2%. In addition, the sample included individuals with a range of functional ability and it was therefore necessary to include a range of intensity levels in order to comprehensively evaluate these monitors in all participants. This resulted in a reduction in the number of participants completing certain activities.

## Conclusion

Movement abnormalities associated with CP may diminish the ability of accelerometry-based devices to estimate EE in this population. Although multi-sensor, accelerometry-based devices have the potential to improve the estimation of EE in people with movement disorders, the results of the current study indicate that a traditional accelerometer provides a more accurate estimate of EE in adults and children with CP. However, all three monitors show large errors for estimating EE and wide limits of agreement. As such, in their current form these monitors should not be used to provide estimates of EE in people with CP. With calibration of CP-specific equations for each level of gross motor function, these monitors may still have the potential to accurately estimate EE in adults and children with CP.

## Abbreviations

CP: Cerebral palsy; EE: Energy expenditure; SWA: Sensewear ProArmband; IDEEA: Intelligent Device for Energy Expenditure and Activity; GMFCS: Gross Motor Function Classification System; IC: Indirect calorimeter.

## Competing interests

The authors declare that they have no competing interests.

## Authors’ contributions

JMR conceptualised and designed the study, collected data, analysed data and drafted the original manuscript. MW contributed to the design of the study and reviewed and revised the manuscript. JG conceptualised and designed the study and reviewed and revised the manuscript. All authors read and approved the final manuscript.
